# Solvation Effects on the Static and Dynamic First-Order Electronic and Vibrational Hyperpolarizabilities of Uracil: A Polarized Continuum Model Investigation

**DOI:** 10.1155/2013/652124

**Published:** 2013-12-22

**Authors:** Andrea Alparone

**Affiliations:** Department of Chemistry, University of Catania, Viale A. Doria 6, Catania 95125, Italy

## Abstract

Electronic (*β* ^*e*^) and vibrational (*β* ^*v*^) first-order hyperpolarizabilities of uracil were determined in gas and water solution using the Coulomb-attenuating Density Functional Theory level with the Dunning's correlation-consistent aug-cc-pVDZ basis set. Frequency-dependent *β* ^*e*^ values were computed for the Second Harmonic Generation (SHG) and Electric Optical Pockels Effect (EOPE) nonlinear optical phenomena. The Polarized Continuum Model was employed to study the solvent effects on the electronic and vibrational properties. The introduction of solvation contributions increases the *β* ^*e*^(static) value by ca. 110%. In comparison, smaller enhancements are found for the *β* ^*e*^(EOPE) and *β* ^*e*^(SHG) data evaluated at the typical wavelength of 694 nm (by 40–50%). The gas-water hyperpolarizability difference was rationalised through a density analysis study. The magnitudes of the vibrational first-order hyperpolarizabilities are comparable to their electronic counterparts and noticeably increase in solution: *β* ^*v*^(EOPE) ~ *β* ^*e*^(EOPE) in aqueous phase at *λ* = 694 nm. Analysis of the IR and Raman spectra is useful to elucidate the most important contributing modes to the vibrational first-order hyperpolarizabilities.

## 1. Introduction

Organic nonlinear optical (NLO) compounds are intensively studied, primarily for their potential use in the design of photonic and optoelectronic devices [1–3]. Biomolecules are attractive NLO materials, having the practical advantage to be already available in nature. Over recent years, DNA-based systems have received great attention for their conductive and NLO applications [4–12]. Nevertheless, characterization of the NLO properties of single nucleic acid bases is still rather incomplete. To the best of our knowledge, experimental response electric properties of the smallest base uracil are not available so far, whereas some theoretical estimates of the electronic polarizabilities (*α* ^*e*^) [13–21] and second-order hyperpolarizabilities (*γ* ^*e*^) [18] were previously reported. However, there is significant interest in exploring the second-order NLO effects, which are important for immediate practical applications. At the microscopic level, the second-order NLO properties are associated with the first-order hyperpolarizability tensor (*β*_*ijk*_), which originates from the responses of a molecular system to external electric field strengths F_i_, producing an induced dipole moment *μ*_*i*_(*F*_*i*_):

(1) $$\begin{array}{c} \mu_{i}\left(F_{i}\right)=\mu_{i}\left(0\right)+\alpha_{ij}F_{j}+\frac{1}{2!}\beta_{ijk}F_{j}F_{k}+\frac{1}{3!}\gamma_{ijkl}F_{j}F_{k}F_{l}+\cdots . \end{array}$$

Recently, pure vibrational contributions to the first-order hyperpolarizability of uracil have been calculated in vacuum through a Lanczos procedure [22], whereas explorations of solvent effects on the electronic and vibrational *β* values are still lacking to date.

Our current computational study mainly focuses on the electronic (*β* ^*e*^) and vibrational (*β* ^*v*^) static and dynamic first-order hyperpolarizabilities of uracil. The fundamental role of the vibrational counterparts to the hyperpolarizabilities has been widely documented [23]. The present calculations were performed in gas and water solution under the Polarized Continuum Model (PCM) approximation [24, 25]. There are many indications in the literature showing that calculated first-order hyperpolarizabilities of organic molecules are strongly affected by solvent contributions [26–33]. Solvation effects on the electronic and vibrational *α* [18, 19] and *γ* [18] values of uracil have been previously explored by means of PCM Hartree-Fock and DFT computations in carbon tetrachloride, acetonitrile, and water solutions.

## 2. Computational Methods

The present calculations were performed in the gas phase and water solution (*ε* = 78.3553) with the Gaussian 09 package [34]. The solvent effects were entirely modelled under the PCM approximation as implemented in the Gaussian 09 program. The geometry of uracil was optimized under the planar *C*_*s*_ symmetry using the CAM-B3LYP functional [35] and the polarised and diffuse Dunning's correlation-consistent aug-cc-pVDZ basis set [36]. The IR and Raman spectra were simulated under the harmonic approximation at the CAM-B3LYP/aug-cc-pVDZ level on the geometries optimized at the same level. The structures are true minima on the potential energy surfaces (no imaginary wavenumbers).

Static *β* ^*e*^ values were calculated at the CAM-B3LYP/aug-cc-pVDZ//CAM-B3LYP/aug-cc-pVDZ level. We selected the CAM-B3LYP functional and aug-cc-pVDZ basis set considering their satisfactory performances in the prediction of the response electric properties of organic compounds, reproducing adequately first-order hyperpolarizabilities obtained using high-level correlated *ab initio* methods and larger basis sets [28, 29, 37–45]. The dynamic electronic first-order hyperpolarizabilities [*β* ^*e*^(−*ω*_*σ*_; *ω*_1_, *ω*_2_)] for the Second Harmonic Generation [SHG, *β* ^*e*^(−2*ω*; *ω*, *ω*)] and Electric Optical Pockels Effect [EOPE, *β* ^*e*^(−*ω*; *ω*, 0)] NLO phenomena were calculated at the CAM-B3LYP/aug-cc-pVDZ level in the *ħω* range 0–0.06563 a.u. The highest *ħω* value corresponds to the wavelength (*λ*) of 694 nm, which is characteristic of the ruby laser.

Static pure vibrational first-order hyperpolarizabilities were obtained at the CAM-B3LYP/aug-cc-pVDZ//CAM-B3LYP/aug-cc-pVDZ level in vacuum and water solution under the double-harmonic approximation (the used symbols have their standard meaning) [23]:

(2) βijkv=[μα]0,0=∑a3N−6((∂μi∂Qa)0(∂αjk∂Qa)0+(∂μj∂Qa)0(∂αik∂Qa)0+(∂μk∂Qa)0(∂αij∂Qa)0)×(ωa2)−1.

By assuming the validity of the infinity frequency approximation [46], the *β* ^*v*^(SHG) and *β* ^*v*^(EOPE) processes are, respectively,

(3) $$\begin{array}{c} \beta_{ijk}^{v}{\left(-2\omega ;\omega ,\omega \right)}_{\omega \to \infty }=0,\\ \beta_{ijk}^{v}{\left(-\omega ;\omega ,0\right)}_{\omega \to \infty }=\frac{1}{3}{\left[\mu \alpha \right]}^{0,0}. \end{array}$$

In this study we report the invariant first-order hyperpolarizabilities (*β*_*vec*⁡_) [47]:

(4) $$\begin{array}{c} \beta_{\mathrm{vec}}=\sqrt{\beta_{x}^{2}+\beta_{y}^{2}+\beta_{z}^{2}}, \end{array}$$

where *β*_*i*_ (*i* = *x*, *z*) is given by *β*_*i*_ = (1/3)∑_*j*=*x*,*y*,*z*_(*β*_*ijj*_ + *β*_*jij*_ + *β*_*jji*_).

Atomic units are used throughout the work. Conversion factor to the SI is: 1 a.u. of *β*(*e* ^3^ *a*_0_^3^ *E*_*h*_^−2^) = 3.206361 × 10^−53^ C^3^m^3^J^−2^.

## 3. Results and Discussion

Table 1 lists the CAM-B3LYP/aug-cc-pVDZ dipole moments. The largest *μ* component lies along the *z*-axis, recovering ca. 96% of the total *μ* value. The gas phase *μ*(CAM-B3LYP/aug-cc-pVDZ) of 4.57 D overestimates by 18% the experimental datum obtained by microwave measurements [*μ*(exp.) = 3.87 D] [48], being in good agreement with the high-level *ab initio* CCSD(T)/aug-cc-pVDZ estimate of 4.33 D (+5.5%) [19]. The introduction of the solvation contributions increases the *μ* value by 1.4 D (+37%), in qualitative consistency with the observed increase of 0.26 D when passing from the gas phase [48] to dioxane solution [49].

Table 1 also includes the static electronic first-order hyperpolarizability tensor components *β*_*ijj*_^*e*^ (*i* = *x*, *z*; *j* = *x*, *y*, *z*) in gaseous and aqueous phases. In gas, *β*_*zxx*_^*e*^ is in absolute value the predominant component (−106.5 a.u.), whereas in water solution the largest components are *β*_*zxx*_^*e*^(−240.5 a.u.) and *β*_*zzz*_^*e*^(262.3 a.u.). When passing from the gas phase to the water solution, the *β*_*x*_^*e*^ value increases by about a factor of two, whereas on the contrary |*β*_*z*_^*e*^| decreases by ca. a factor of two. From the present computations, *β*_*x*_^*e*^ dominates the first-order hyperpolarizability of both the gaseous and aqueous phases, giving ca. 85% and 99% of the *β*_*vec*⁡_^*e*^ value, respectively.

In order to clarify the solvation effects on the response electric properties, we determined the spatial contributions of electrons to the first-order hyperpolarizabilities by computing density of hyperpolarizability amplitudes, *ρ*_*jk*_^(2)^(*r*) [50, 51]. The *ρ*_*jk*_^(2)^(*r*) is defined as derivative of the charge density function *ρ*(*r*, *F*) with respect to applied electric field strengths *F* (*r* is the position vector). The *ρ*(*r*, *F*) is usually expanded in powers of *F*:

(5) ρ(r,F)=ρ(0)(r)+∑jρj(1)(r)Fj+12!∑jρjk(2)(r)FjFk +12!∑jρjkl(3)(r)FjFkFl+⋯,ρjk(2)(r)=∂2ρ(r,F)∂Fj∂Fk|Fj=0,Fk=0,βijke=−12!∫rρjk(2)(r)dr.

**Figure 1:**
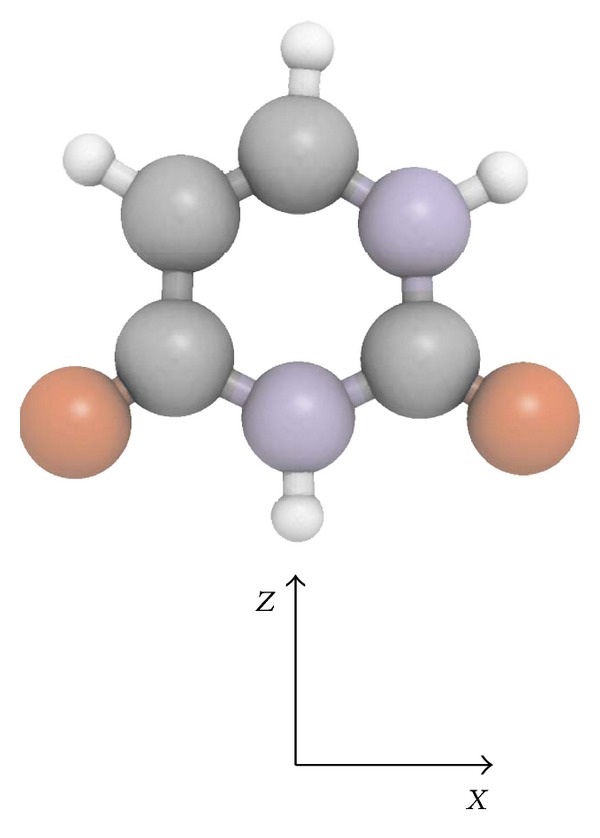
Structure of uracil and Cartesian coordinate system. Colours: white (hydrogen), grey (carbon), red (oxygen), and cyan (nitrogen) (colour figure online).

**Figure 2:**
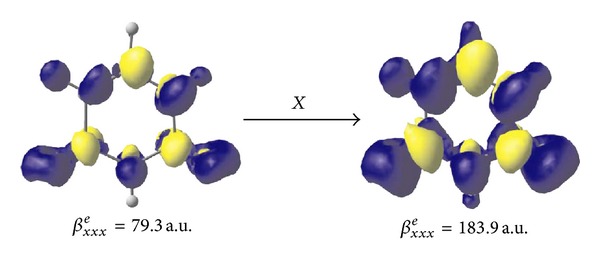
Hyperpolarizability density distributions *ρ*_*xx*_^(2)^(*r*) of uracil in gas (left) and water solution (right). The yellow and blue surfaces (colour figure online) refer to positive and negative *ρ*_*xx*_^(2)^(*r*) densities, respectively, computed at the isosurface of 0.25 a.u. CAM-B3LYP/aug-cc-pVDZ results.

For a certain positive-negative *ρ*_*jk*_^(2)^(*r*) pair, the sign is positive when the direction of the positive to negative density is coincident with the positive direction of the chosen coordinate system (Figure 1), whereas the magnitude is proportional to the distance between the two densities. Following the current calculations, the main contribution to *β*_*x*_^*e*^ is given by the *β*_*xxx*_^*e*^ component, recovering ca. 75% (64%) and 73% (72%) of the *β*_*x*_^*e*^(*β*_*vec*⁡_^*e*^) values in gas and water solution, respectively. Therefore, we explored the *ρ*_*xx*_^(2)^(*r*) densities at the CAM-B3LYP/aug-cc-pVDZ level using the numerical procedure previously illustrated by Yamada and coworkers [51]. The results evaluated at the isosurface of 0.25 a.u. are illustrated in Figure 2. As can be appreciated from the graphical representations, the *ρ*_*xx*_^(2)^(*r*) distribution in the water solution is almost similar to that predicted in vacuum even if the amplitudes are much more spread out. This result is in some consistency with the calculated static *β*_*xxx*_^*e*^ values, with the *β*_*xxx*_^*e*^(water)/*β*_*xxx*_^*e*^(gas) and *β*_*vec*⁡_^*e*^(water)/*β*_*vec*⁡_^*e*^(gas) ratios being computed to be 2.3 and 2.1, respectively. Note that the above ratios are somewhat greater than those previously predicted for the average *α* ^*e*^(1.3) and *γ* ^*e*^(1.5) properties by HF/aug-cc-pVDZ computations [18].

**Figure 3:**
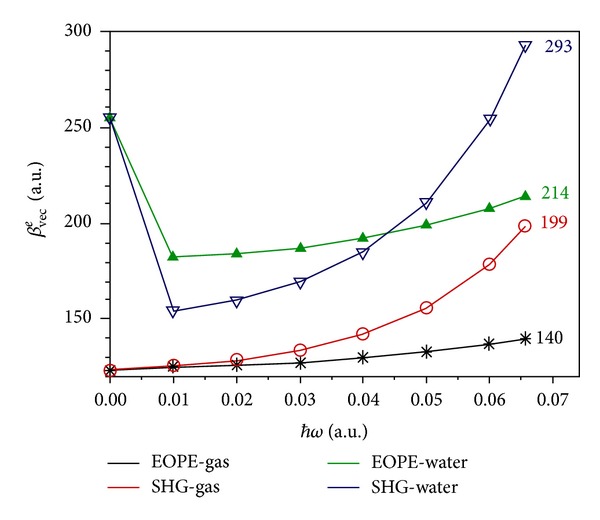
Frequency-dependent electronic first-order hyperpolarizability of uracil in gas and water solution. CAM-B3LYP/aug-cc-pVDZ results. The reported data refer to the *β*_*vec*⁡_^*e*^(−*ω*_*σ*_; *ω*_1_, *ω*_2_) values obtained at *ħω* = 0.06563 a.u.

Figure 3 displays the frequency-dependent first-order hyperpolarizabilities computed in gaseous and aqueous phases in the 0–0.06563 *ħω* range for the SHG and EOPE NLO processes. It is important to notice that resonance enhancement effects for the SHG phenomenon are expected to be rather marginal, since the experimental lowest-energy absorption being placed at 5.08 eV (0.1867 a.u.) in vapour [52] and 4.77 eV (0.1753 a.u.) in water solution [53] is sufficiently far from the highest 2 *ħω* value of 0.1307 a.u. Not surprisingly, in the gas phase *β*_*vec*⁡_^*e*^(−2*ω*; *ω*; *ω*) > *β*_*vec*⁡_^*e*^(−*ω*; *ω*; 0) > *β*_*vec*⁡_^*e*^(0; 0; 0). The dispersion effects evaluated at the *ħω* = 0.06563 a.u. increase the static values by 13.7% for the EOPE and by 61.5% for the SHG process. On the other hand, in aqueous phase the dispersion effects are significantly reduced, mainly due to incomplete responses of polar solvents as modelled by the PCM treatment [31–33, 54]. As can be appreciated from Figure 3, in water solution *β*_*vec*⁡_^*e*^(−2*ω*; *ω*; *ω*)<*β*_*vec*⁡_^*e*^(−*ω*; *ω*; 0) for the *ħω* values between 0.01 and 0.04 a.u., whereas *β*_*vec*⁡_^*e*^(−2*ω*; *ω*; *ω*) ~ *β*_*vec*⁡_^*e*^(−*ω*; *ω*; 0) at *ħω*∼ 0.045 a.u. It is worth noting that at the *ħω* value of 0.06563 a.u. the dispersion effect is negative for the EOPE phenomenon, with the *β*_*vec*⁡_^*e*^(−*ω*; *ω*; 0)(water) value being decreased by ca. 16.0% with respect to the static datum. On the other hand, in the case of the SHG process in water solution, the dispersion effect at *ħω* = 0.06563 a.u. is still positive as for the gas phase, even if it is noticeably inferior (+15.5%). As a consequence, although the static and dynamic electronic first-order hyperpolarizabilities in water solution are greater than the corresponding data in gas (compare the curves in Figure 3), the dispersion effects reduce the *β*_*vec*⁡_^*e*^(water)/*β*_*vec*⁡_^*e*^(gas) ratios, which are predicted to be 2.1, 1.53 and 1.48, respectively, for the static, EOPE, and SHG processes at *ħω* = 0.06563 a.u.

**Figure 4:**
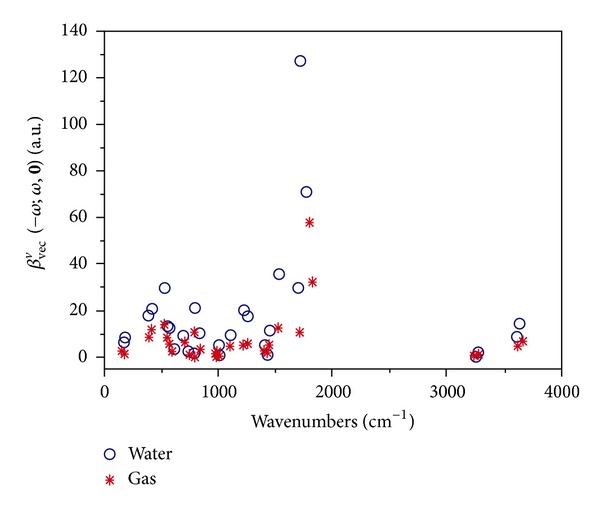
Contribution of each normal mode to the vibrational first-order hyperpolarizability of uracil in gas and water solution. CAM-B3LYP/aug-cc-pVDZ results.

**Figure 5:**
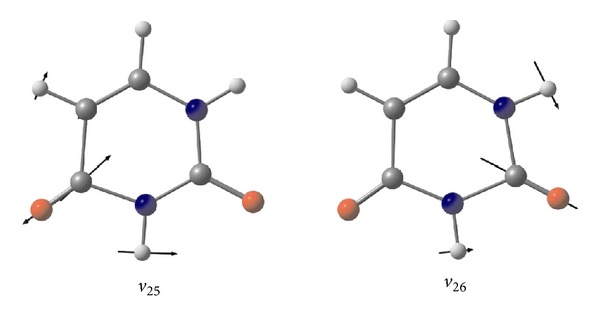
Atom vector displacements of the *ν* C = O +  *δ*N-H modes *ν*_25_ and *ν*_26_.

**Figure 6:**
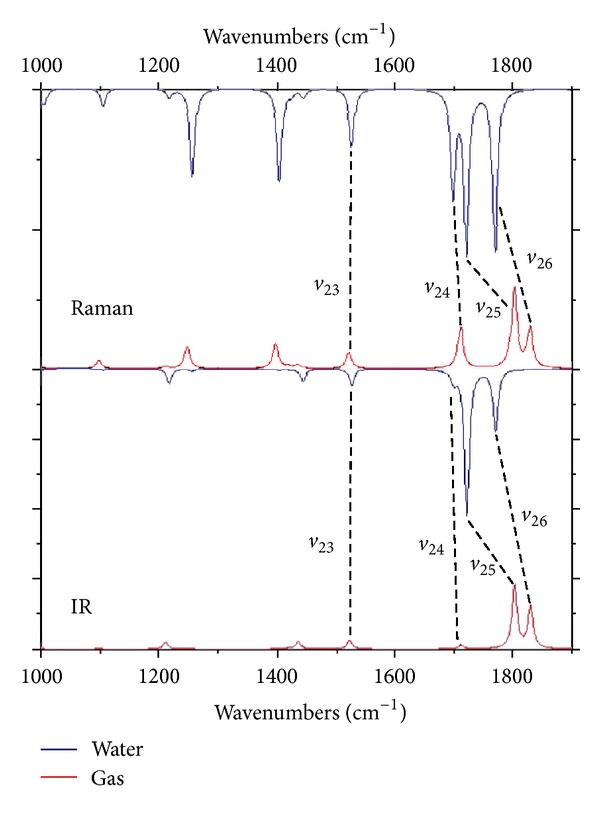
IR and Raman spectra of uracil in gas and water solution in the 1000–1900 cm^−1^ wavenumbers range. Lorentz line shapes with a full width at half maximum of 10 cm^−1^ were used. CAM-B3LYP/aug-cc-pVDZ results.

Beside the electronic first-order hyperpolarizability, we explored the solvation effects on the pure vibrational counterpart for the EOPE phenomenon. In a recent theoretical study, Christiansen and coworkers have determined the pure vibrational first-order hyperpolarizabilities of uracil in gas using VCI computations and the Lanczos algorithm [22]. However, their reported data refer to the static and SHG process and are not directly comparable to our results.  Figure 4 shows the CAM-B3LYP/aug-cc-pVDZ *β*_*vec*⁡_^*v*^(−*ω*; *ω*; 0) data in gaseous and aqueous phases over the 0–4000 cm^−1^ wavenumbers range. The largest contributions originate from the spectral region between 1500 and 2000 cm^−1^.  Table 2 summarizes the main vibrational contributions to the *β*_*vec*⁡_^*v*^(−*ω*; *ω*; 0) values, also including the vibrational wavenumbers, IR intensities (*I*_IR_), and Raman activities (*A*_Raman_). The highest-energy region (wavenumbers >3000 cm^−1^), entirely characterized by C–H and N–H stretching vibrations, furnishes only modest contributions to the *β*_*vec*⁡_^*v*^(−*ω*; *ω*; 0) data, principally owing to the high-wavenumber values. On the other hand, the low-energy modes (wavenumbers <1000 cm^−1^) produce small and moderate *β*_*vec*⁡_^*v*^(−*ω*; *ω*; 0) contributions, since the vibrational transitions are rather weak in the IR [*I*_IR_∝(∂*μ*_*i*_/∂*Q*_*a*_)^2^] and Raman [*A*_Raman_∝ (∂*α*_*i*_/∂*Q*_*a*_)^2^] spectra. As for the pure vibrational polarizabilities [19], in both the gas phase and water solution, the largest *β*_*vec*⁡_^*v*^(−*ω*; *ω*; 0) values originate from the C=O stretching vibrations with the nonnegligible contribution of the in-plane N-H bending deformation (modes *ν*_25_ and *ν*_26_). A graphical representation of the atomic displacement vectors involved in these vibrational modes is displayed in Figure 5. These transitions located at 1802 cm^−1^ (*ν*_25_) and 1828 cm^−1^ (*ν*_26_) by the present calculations in vacuum exhibit the strongest absorption peaks in the IR spectrum (Figure 6), with the *I*_IR_ values of 902 and 607 km/mol, respectively. Note that the *ν*_25_ and *ν*_26_ modes are also active in the Raman spectra (*A*_Raman_ = 58 and 29 Å^4^/amu, resp.). As a result, the *β*_*vec*⁡_^*v*^(−*ω*; *ω*; 0) values originated by the *ν*_25_ and *ν*_26_ transitions contribute, respectively, to ca. 89% and 50% of the total *β*_*vec*⁡_^*v*^(−*ω*; *ω*; 0) datum. In water solution (Figure 6), the wavenumbers of the *ν*_25_ and *ν*_26_ vibrations are downward shifted, respectively, by ca. 80 cm^−1^ (−4.5%) and 60 cm^−1^ (−3.2%) with respect to the gas phase, with the *I*_IR_ values being concomitantly increased by ca. 131% and 45%, respectively. In addition, when passing from the gaseous to the aqueous phase, the *A*_Raman_(*ν*_25_) and *A*_Raman_(*ν*_26_) values enhance by ca. a factor of two and four, respectively. Therefore as for the *β* ^*e*^ values, the solvent contributions are expected to play a crucial role also for the vibrational first-order hyperpolarizabilities of uracil, increasing the *β*_*vec*⁡_^*v*^(−*ω*; *ω*; 0) values of the *ν*_25_ and *ν*_26_ modes by 69.4 a.u. (+120%) and 38.5 a.u. (+119%). As can be appreciated by the data reported in Table 2, for both the phases other relevant *β*_*vec*⁡_^*v*^(−*ω*; *ω*; 0) contributions are given by the ring stretching modes as well as by the in-plane ring bending deformations, owing to their relatively low wavenumbers and moderate *I*_IR_ and *A*_Raman_ values. On the whole, the introduction of solvent contributions increases the total *β*_*vec*⁡_^*v*^(−*ω*; *ω*; 0) by ca. 130 a.u., with the *β*_*vec*⁡_^*v*^(−*ω*; *ω*; 0)(water)/*β*_*vec*⁡_^*v*^(−*ω*; *ω*; 0)(gas) ratio being predicted to be ca. three. Finally, it is worth noting that, at *ħω* = 0.06563 a.u. on going from the gas phase to water solution, the *β*_*vec*⁡_^*v*^(−*ω*; *ω*; 0)/*β*_*vec*⁡_^*e*^(−*ω*; *ω*; 0) ratio is almost doubled, increasing from 0.46 to 0.91.

## 4. Conclusions

We have examined the effects of solvation on the static and frequency-dependent electronic and vibrational first-order hyperpolarizabilities of uracil. The properties were modeled in vacuum as well as in water solution using the PCM approach. The calculations were carried out using the long-range corrected CAM-B3LYP functional with the Dunning's correlation-consistent aug-cc-pVDZ basis set. The introduction of solvent contributions significantly increases both the electronic and vibrational first-order hyperpolarizabilities. However, the dispersion effects on the electronic hyperpolarizabilities for the EOPE and SHG NLO phenomena are noticeably reduced when passing from the gas phase to the water solution. The magnitudes of the vibrational properties are comparable to the electronic counterparts, with the *β*_*vec*⁡_^*v*^/*β*_*vec*⁡_^*e*^ ratio increasing with the solvation and *β*_*vec*⁡_^*v*^(water)∼*β*_*vec*⁡_^*e*^(water) for the EOPE process at the characteristic wavelength of 694 nm. The most relevant contributing modes to the *β* ^*v*^ values principally involve the very intense infrared C=O stretching + N-H in-plane bending deformation vibrations.

## Figures and Tables

**Table 1:** Dipole moments *μ* (D) and static electronic first-order hyperpolarizabilities *β* ^*e*^ (a.u.) of uracil^a^.

	Gas	Water
*μ*_*x*_	1.21	1.88
*μ*_*z*_	4.41	5.96
*μ*	4.57 (3.87)^b^	6.25
*β*_*xxx*_^*e*^	79.3	183.9
*β*_*xyy*_^*e*^	19.5	44.0
*β*_*xzz*_^*e*^	5.7	24.6
*β*_*zxx*_^*e*^	−106.5	−240.5
*β*_*zyy*_^*e*^	−36.5	−56.4
*β*_*zzz*_^*e*^	78.2	262.3
*β*_x_^e^	104.6	252.5
*β*_z_^e^	−64.8	−34.6
*β*_*vec*⁡_^e^	123.0	254.9

^a^Calculations were carried out at the CAM-B3LYP/aug-cc-pVDZ  level on the geometry calculated at the same level.

^b^Reference [48].

**Table 2:** Selected vibrational contributions to the first-order hyperpolarizabilities of uracil^a^.

	Mode no.	Wavenumbers (cm^−1^)	*I*_IR_ (km/mol)	A_Raman _(Å^4^/amu)	Description^b^	*β*_*vec*⁡_^e^ (−*ω*; *ω*, **0**) (a.u.)^c^
Gas	*ν*_4_	411	20	1	*τ*ring	11.5
	*ν*_5_	524	22	2	*δ*ring	13.8
	*ν*_11_	783	4	22	*δ*ring	10.6
	*ν*_23_	1523	127	11	*ν*ring + *δ*N-H	12.5
	*ν*_24_	1711	56	30	*ν*ring + *δ*C-H	10.4
	*ν*_25_	1802	902	58	*ν*C=O + *δ*N-H	57.8
	*ν*_26_	1828	607	29	*ν*C=O + *δ*N-H	32.4
					Total	**65.0 (139.8)** ^ d^

Water	*ν*_5_	530	45	4	*δ*ring	29.5
	*ν*_23_	1527	236	42	*ν*ring + *δ*N-H	35.6
	*ν*_24_	1699	159	75	*ν*ring + *δ*C-H	29.6
	*ν*_25_	1721	2086	115	*ν*C=O + *δ*N-H	127.2
	*ν*_26_	1769	877	116	*ν*C=O + *δ*N-H	70.9
					Total	**193.9 (214.1)** ^ d^

^a^Calculations were carried out at the CAM-B3LYP/aug-cc-pVDZ level on the geometry calculated at the same level. The contributions with percentage ≥15% of the total *β*_*vec*⁡_^*v*^ (−*ω*; *ω*, **0**) value were considered.

^b^ *v*: stretching, *δ*: in-plane bending, *τ*: torsion.

^c^The value in parentheses refers to the CAM-B3LYP/aug-cc-pVDZ *β*_*vec*⁡_^e^ (−*ω*; *ω*, **0**) value at *ħω* = 0.06563 a.u.

^d^The β_*vec*⁡_^*v*^ (−ω; ω, 0)/β_*vec*⁡_^*e*^ (−ω; ω, 0) ratios are 0.46 and 0.91 in gas and water solution, respectively.
